# Rumination and alexithymia serially mediate the relationship between mindfulness and anxiety symptoms in Chinese university students

**DOI:** 10.1038/s41598-025-11973-0

**Published:** 2025-07-16

**Authors:** Junchao Yuan, Luyu Chen, Hongye Geng

**Affiliations:** School of Teacher Education, Xichang University, Xichang, 615013 Sichuan China

**Keywords:** Mindfulness, Anxiety symptoms, Rumination, Alexithymia, University students, Mediation analysis, Psychology, Health care

## Abstract

Anxiety symptoms are highly prevalent among Chinese university students, significantly impairing psychosocial functioning. Although mindfulness has been identified as a protective psychological factor, the precise mechanisms underlying its association with anxiety symptom alleviation remain insufficiently understood. This study investigates the serial mediation effects of rumination and alexithymia in the relationship between mindfulness and anxiety symptoms. A cross-sectional study was conducted with 860 Chinese university students. Validated psychometric instruments were administered, including the Mindful Attention Awareness Scale (MAAS), Ruminative Response Scale (RRS), Toronto Alexithymia Scale (TAS-20), and Screen for Adult Anxiety Related Disorders (SCAARED). Serial mediation analyses were performed using SPSS 25.0 and Hayes’ PROCESS macro (Model 6), controlling for sex and academic grade. Higher mindfulness levels showed robust associations with lower anxiety symptom severity. This relationship was primarily linked to interconnected cognitive and affective pathways. The analysis revealed that individuals reporting higher mindfulness exhibited lower tendencies toward repetitive negative thinking (rumination), which corresponded to lower anxiety levels. Simultaneously, higher mindfulness was associated with lower alexithymia that typically co-occur with anxiety symptoms. Crucially, a sequential pathway emerged: higher mindfulness correlated with lower rumination, which in turn corresponded to fewer emotional processing difficulties (alexithymia), ultimately associating with fewer anxiety symptoms. Collectively, these indirect pathways accounted for most of mindfulness’s observed relationship with anxiety symptoms, highlighting interconnected roles of cognitive and emotional factors. Mindfulness shows protective associations with anxiety symptoms through dual correlations: correspondence with lower rumination and relationship with lower alexithymia. These findings advocate for interventions addressing both cognitive patterns and emotional regulation in culturally adapted campus mental health programs.

**Supplementary Information:**

The online version contains supplementary material available at 10.1038/s41598-025-11973-0.

## Introduction

Anxiety symptoms represent a global public health challenge, ranking among the most prevalent mental health conditions worldwide. These symptoms are characterized by persistent and excessive worry, heightened sensitivity to uncertainty, and substantial functional impairments across quality of life, academic achievement, and social relationships^1,2^. Epidemiological data from the World Health Organization reveal a disproportionate disease burden in low- and middle-income countries, exacerbated by the chronic course of anxiety symptoms and their frequent comorbidity with depression and other psychiatric conditions^3–5^. Particularly concerning is the escalating prevalence among university populations, with recent studies attributing this trend to multifactorial stressors including academic pressures, social adaptation challenges, career competition, and interpersonal difficulties^6^. Contemporary research indicates lifetime and 12-month prevalence rates of Generalized Anxiety Disorder (GAD) in university students at 18.6% and 16.7%, respectively^7^.

The Chinese context presents unique epidemiological characteristics, with anxiety symptoms rates among university students substantially exceeding those observed in Western populations. A multinational meta-analysis by during the COVID-19 pandemic university students reported a pooled anxiety prevalence of 41% (95% CI: 0.34–0.49), with the Asian subgroup at 33% (95% CI: 0.25–0.43)^8^. Longitudinal evidence from Gao et al.revealed > 45% of female and > 40% of male Chinese freshmen demonstrated clinically significant anxiety^9^. Although prevalence decreased in senior years (~ 38%), overall rates remained elevated. A systematic review of 76 studies further confirmed a merged anxiety symptom prevalence of 39.0% (95% CI: 34.6–43.4%) in this population^10^. A nationwide 2022 survey revealed that 45.28% of Chinese university students met clinical thresholds for anxiety disorder risk, a rate significantly higher than both the general Chinese adult population and international student cohorts^11^. This disparity has been linked to enduring academic pressures from China’s competitive education system - including residual effects of the college entrance examination (Gaokao) and academic burnout - coupled with the psychosocial impacts of rapid digital transformation in social communication and reflecting a global public health crisis intensified among Chinese university students post-COVID-19.

Current therapeutic approaches primarily utilize pharmacological interventions (e.g., selective serotonin reuptake inhibitors) and psychological modalities such as cognitive-behavioral therapy (CBT). However, clinical implementation faces significant barriers including medication side effects, treatment accessibility issues, and suboptimal adherence rates^12^. These limitations underscore the critical need for developing cost-effective, low-risk interventions with enhanced scalability.

Emerging evidence positions mindfulness-based interventions (MBIs) as a promising therapeutic alternative. Defined as the nonjudgmental awareness of present-moment experiences, mindfulness demonstrates robust inverse correlations with anxiety severity across clinical and non-clinical populations^13^. The mindfulness reperceiving model proposes that mindfulness facilitates anxiety reduction through two primary mechanisms: (1) enhanced metacognitive awareness enabling objective reappraisal of emotional experiences, and (2) disruption of maladaptive cognitive-emotional patterns through decentering from automatic reactivity. Nevertheless, critical gaps persist in understanding the precise psychological mechanisms underlying these effects, particularly regarding the dynamic interplay between cognitive reappraisal processes and emotional regulation pathways^14^.

### Mindfulness in the context of anxiety symptoms

Mindfulness meditation demonstrates therapeutic efficacy in addressing psychological and physiological conditions, including pain, anxiety symptoms, and eating pathologies^15^. As a lifestyle-oriented intervention, it provides cost-effective therapeutic benefits, enhances clinical prognoses, and exhibits no documented adverse effects^16^. Empirical studies confirm its capacity to significantly reduce anxiety symptoms across both non-clinical and clinical cohorts^17,18^. A randomized controlled trial (RCT) conducted with Chinese university students revealed substantial anxiety symptom reduction following an 8-week mindfulness intervention^19^. A meta-analysis of 55 RCTs further substantiated these findings, reporting a moderate-to-large pooled effect size for anxiety reduction (g = 0.60) associated with mindfulness-based interventions^20^.

Despite established inverse correlations between mindfulness and anxiety symptoms severity, the mediating mechanisms remain insufficiently elucidated. Specifically, the potential serial mediation through cognitive and emotional factors—particularly the temporal sequencing of these processes—requires rigorous empirical investigation.

### The potential mediating role of rumination

Rumination represents a maladaptive cognitive pattern marked by persistent, uncontrollable engagement with negative ideation, a process recognized for exacerbating anxiety symptomatology^21^. As a prominent cognitive risk factor in anxiety etiology, rumination has emerged as a critical focus of empirical inquiry. Neurocognitive research indicates that chronic negative thought patterns amplify attentional bias toward perceived threats, thereby potentiating the intensity and duration of anxious states^22^.

Mindfulness-based practices have been identified as effective interventions for disrupting cyclical negative cognition and attenuating ruminative tendencies^23,24^. Pathological rumination demonstrates multifaceted cognitive consequences, compromising executive function, enhancing accessibility of negative autobiographical memories, diminishing recognition of positively valenced stimuli, and fostering attentional prioritization of negative emotional cues^25,26^. These cognitive alterations collectively implicate rumination as a mechanistic contributor to both anxiety symptoms pathogenesis and emotional dysregulation.

Current literature has predominantly examined rumination’s independent mediation effects, while a critical gap remains in elucidating its synergistic interactions with affective regulatory processes in shaping anxiety symptoms trajectories.

### The potential mediating role of alexithymia

Emotion Regulation Theory posits that individuals modulate and modify emotional experiences through both conscious and unconscious regulatory strategies^27^. Impairments in emotion regulation capacities—particularly deficits in emotional awareness and expressive abilities—compromise adaptive management of negative affect, thereby elevating vulnerability to anxiety symptoms^28^.

Alexithymia, a multidimensional deficit in emotional processing marked by impaired emotional identification and verbal expression, constitutes a pathogenic mechanism in anxiety symptoms pathogenesis^29^. This construct demonstrates robust associations with anxiety severity, particularly among populations exhibiting compromised emotional recognition and descriptive capacity^30^. Empirical evidence indicates that mindfulness training enhances emotional granularity and nonjudgmental acceptance, facilitating accurate emotional identification and expression, which consequently reduces alexithymic traits and attenuates anxiety symptom severity^31^. Neurocognitive research has established emotion regulation as a critical mediating mechanism linking mindfulness practices to anxiety reduction^32^. Notably, individuals with elevated dispositional mindfulness exhibit enhanced regulatory resilience during recovery from negative emotional states^33^. Mindfulness-based interventions further demonstrate therapeutic efficacy in improving emotional clarity, thereby ameliorating the psychopathological consequences of alexithymia^34^.

However, the potential moderation of this mediational pathway by co-occurring cognitive processes—particularly the interaction between alexithymia and maladaptive rumination patterns—remains insufficiently characterized.

### The potential serial mediating role of rumination and alexithymia

Rumination and alexithymia may constitute critical mediating pathways in the mindfulness-anxiety symptoms association^35,36^. The cognitive-emotional processing model posits that chronic rumination compromises emotion regulation capacity, thereby potentiating both alexithymic traits and anxiety symptomatology^37^. Emerging theoretical frameworks suggest that rumination may indirectly correlates with alexithymia through impaired emotional regulation, establishing a maladaptive “cognitive fixation-emotional blunting” cycle^38^. Persistent cognitive engagement with negative stimuli disrupts accurate emotional perception and expression, amplifying emotional distress through dual cognitive-affective pathways^39,40^. Mindfulness-based practices may alleviate anxiety symptoms via synergistic enhancement of cognitive flexibility and emotional regulatory competence^41,42^.

Although rumination and alexithymia have long been considered mutually influential constructs, emerging evidence suggests rumination may serve as an antecedent to alexithymia development. Persistent rumination potentially impairs emotional differentiation and expression capabilities, thereby contributing to alexithymia onset. Bal and Alkoç demonstrated that negative automatic thoughts and rumination significantly predict alexithymia emergence, particularly during experiences of cognitive dissonance and helplessness^43^. This aligns with cognitive theories regarding rumination’s role in emotional processing deficits.

Emotion Regulation Theory emphasizes cognitive strategies’ active modulation of emotional responses, positioning rumination as a passive, maladaptive response-focused strategy that exacerbates negative affect^27^. The Emotional Cascade Theory proposes that rumination interacts with emotion identification difficulties (core to alexithymia) to promote dysregulated behaviors^44^. Specifically, rumination depletes cognitive resources and compromises emotion recognition capacity (i.e., alexithymia), mediating the relationship between emotion regulation difficulties and alexithymia. This framework underscores rumination’s pivotal role in emotional disorder pathogenesis.

The Response Styles Theory conceptualizes rumination as passive, repetitive focus on depressive symptoms that impedes problem-solving and prolongs negative states^39^. Consequently, individuals with high rumination tendencies demonstrate impaired access to adaptive regulatory strategies, leading to cumulative emotional dysregulation.

Existing research predominantly employs single-mediation models to examine mindfulness-anxiety relationships, overlooking dynamic cross-system interactions between cognitive and affective mechanisms. Although rumination and alexithymia are independently validated as anxiety risk factors, their reciprocal cognitive interplay remains insufficiently mapped.

### The current study

This study empirically adjudicates between these competing frameworks.The current study examine the mechanistic pathways through which mindfulness influences anxiety symptoms in Chinese university students, with specific focus on rumination and alexithymia as serial mediators. To our knowledge, this represents the first empirical effort to concurrently model cognitive and affective mediation processes in this context. The study aims to advance theoretical frameworks for mindfulness-based anxiety interventions while evaluating the cross-cultural applicability of this model within China’s unique academic environment, thereby providing actionable insights for culturally tailored mental health strategies.

**Fig. 1:**
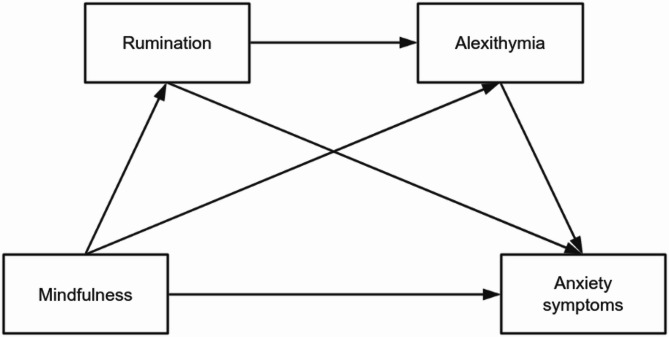
Hypothesized serial mediating model.

Whether rumination and alexithymia operate sequentially or reciprocally thus constitutes a pivotal theoretical contention. Guided by the aforementioned theoretical synthesis, we propose a serial mediation model (shown in Fig. 1) and propose the following four hypotheses: (1)Mindfulness exhibits significant negative associations with anxiety symptoms severity.(2)Rumination independently mediates the mindfulness-anxiety symptoms relationship.(3)Alexithymia independently mediates the mindfulness-anxiety symptoms relationship.(4)Rumination and alexithymia jointly form a serial-mediating pathway between mindfulness and anxiety symptoms.

While our model hypothesizes rumination co-occurs with alexithymia in a sequential pattern, alternative theoretical frameworks propose reversed or reciprocal associations. For instance, Emotion Regulation Theory suggests that deficits in emotional awareness (alexithymia) may correlate with maladaptive rumination, concurrent with impaired cognitive resolution of distress^27^. Further, the Emotional Cascade Theory implies bidirectional covariance between these constructs^44^. These perspectives are not mutually exclusive; our proposed sequence represents one cognitively oriented association supported by evidence that rumination correlates with reduced availability of executive resources required for emotional differentiation^37,38^. The current study statistically evaluates these alternatives through competing mediation models.

## Method

### Participants and recruitment

This cross-sectional study analyzed data from a mental health survey conducted at a public university in Southwest China. Data collection was conducted during spring 2024 using a certified web-based platform adhering to ISO 27,001 information security standards.

A pilot study (*n* = 50) preceded full-scale implementation to verify instrument reliability and procedural feasibility. Participants independently completed the anonymous questionnaire within a 20-minute timeframe, with confidentiality assurances and explicit academic purpose declarations to mitigate social desirability biases.

Using non-probability convenience sampling, we distributed questionnaires to freshmen through seniors. From 995 initial responses, 860 met inclusion criteria after excluding 135 submissions with patterned responses (≥ 10 consecutive identical items) or logical inconsistencies (e.g., denying anxiety while endorsing panic symptoms). The final analytic sample demonstrated an 86.4% validity rate.

Sex refers to birth-assigned biological status (male/female), consistent with NIH SAGER Guidelines. Gender identity was not assessed due to scale limitations. Demographic characteristics revealed female predominance (85.5%, *n* = 735) versus male participants (14.5%, *n* = 125). Class distribution comprised freshmen (46.5%, *n* = 400), sophomores (24.9%, *n* = 214), juniors (23.3%, *n* = 200), and seniors (5.3%, *n* = 46). These population characteristics were subsequently treated as covariates in multivariable analyses to control for potential confounding effects.

This study was conducted in accordance with the Declaration of Helsinki and approved by the Ethics Committee of Xichang University (Approval No. XCUEC2024-03). All participants provided written informed consent before participating in the survey.

### Measures

#### Mindful attention awareness scale

Mindfulness was assessed using the 15-item Mindful Attention Awareness Scale (MAAS), adapted into Chinese by Chen et al.^45,46^. This unidimensional scale measures the frequency of automatic and unaware behaviors in daily life. Items are rated on a 6-point Likert scale from 1 (“almost always”) to 6 (“almost never”), with higher total scores reflecting greater mindfulness and present-moment awareness. In this study, the scale demonstrated good internal consistency (Cronbach’s *α* = 0.895).

#### Screen for adult anxiety related disorders

Anxiety disorders were assessed using the Chinese version of the Screen for Adult Anxiety Related Disorders (SCAARED)^47,48^. The 44-item scale evaluates symptoms across four domains: generalized anxiety disorder, social anxiety disorder, panic disorder, and separation anxiety disorder. Responses are recorded on a 3-point scale (0 = “never”, 1 = “sometimes”, 2 = “often”), with total scores ranging from 0 to 88. A cutoff score of ≥ 23 indicates clinically significant anxiety symptoms. The scale showed excellent reliability in this sample (Cronbach’s *α* = 0.94).

#### Ruminative responses scale

Rumination was measured with the Ruminative Responses Scale (RRS), validated in Chinese by Han et al.^49,50^. The 22-item scale comprises three subscales: symptom-focused rumination, brooding, and reflective pondering. Participants rate items on a 4-point Likert scale from 1 (“never”) to 4 (“always”), with higher scores indicating stronger rumination tendencies. Internal consistency in the current study was high (Cronbach’s *α* = 0.90).

#### Toronto alexithymia scale

Alexithymia was assessed using the Toronto Alexithymia Scale-20 (TAS-20), adapted for Chinese populations by Yi et al.^51,52^. The 20-item scale includes three subdimensions: difficulty identifying feelings (DIF), difficulty describing feelings (DDF), and externally oriented thinking (EOT). Items are rated on a 5-point Likert scale (1 = “strongly disagree” to 5 = “strongly agree”), with total scores categorized as non-alexithymia (≤ 51), borderline (52–60), or clinical alexithymia (≥ 61). The scale exhibited acceptable reliability (Cronbach’s *α* = 0.83).

### Statistical analyses

All analyses were conducted using SPSS 25.0 with Hayes’ PROCESS macro (v4.3) for mediation modeling, implementing sequential analytical procedures to ensure methodological rigor.

Harman’s single-factor test was first employed to evaluate common method variance, with the critical threshold set at < 40% explained variance for the first unrotated factor.

Preliminary analyses then assessed variable distributions through descriptive statistics (skewness and kurtosis) and confirmatory normality tests (Kolmogorov-Smirnov and Shapiro-Wilk), where absolute skewness (< 3) and kurtosis (< 10) values were evaluated against robust maximum likelihood estimation thresholds while invoking central limit theorem applicability for large samples to justify parametric analyses despite statistically significant normality test results.

Subsequent analyses characterized demographic patterns using independent samples t-tests for sex differences and one-way ANOVA for grade-level comparisons, while bivariate relationships were examined through Pearson correlation matrices with multicollinearity diagnostics (VIF < 3).

The core analysis tested the hypothesized serial mediation pathway (mindfulness → rumination → alexithymia → anxiety) using PROCESS Model 6, with sex dummy-coded (0 = male; 1 = female) and grade mean-centered as covariates, employing bias-corrected bootstrapping with 5,000 resamples to determine statistical significance of indirect effects (95% CIs excluding zero indicating significance).

Finally, to address theoretical concerns regarding mediator directionality, three competing models were validated: the original sequence (Model A: mindfulness → rumination → alexithymia → anxiety), reversed sequence (Model B: mindfulness → alexithymia → rumination → anxiety), and parallel mediation (Model C: mindfulness → [rumination + alexithymia] → anxiety), evaluated using PROCESS Model 6 (serial) and Model 4 (parallel) with identical covariates and resampling procedures, assessing model adequacy through explained variance (*R²*), significance of indirect effects/paths, and theoretical parsimony to ensure comprehensive statistical robustness.

Supplementary analyses including normality diagnostics and competing mediation models are available via the journal’s Electronic Supplementary Materials portal.

## Results

### Common method Bias assessment

Given the study’s sole reliance on self-report data, we conducted Harman’s single-factor test to assess potential common method variance^53^. An unrotated principal component analysis revealed 10 factors with eigenvalues exceeding 1.0. The first factor accounted for 35.09% of the total variance, which falls below the 40% critical threshold established by Podsakoff et al. for significant method bias^54^. This pattern demonstrates that common method variance did not substantially influence the observed relationships.

### Descriptive statistics and bivariate correlations

Psychometric evaluation revealed clinically significant anxiety symptoms (SCAARED: M = 25.69 ± 17.26, range = 3–88), with 49.19% of participants (*n* = 423) surpassing clinical thresholds (> 23). Concurrently, 18.60% (*n* = 160) exhibited diagnostic-level alexithymia (TAS-20: M = 53.29 ± 9.89, range = 28–85; cutoff ≥ 61). As detailed in Table 1, supplementary measures displayed characteristic distributions: mindfulness (MAAS: M = 57.22 ± 15.80) and rumination (RRS: M = 39.34 ± 12.95).

Preliminary analyses assessed the normality of all study variables (*N* = 860) using descriptive statistics (skewness and kurtosis) and confirmatory statistical tests (Kolmogorov-Smirnov and Shapiro-Wilk). As reported in Supplementary Table S1, all variables exhibited statistically significant deviations from normality on both Kolmogorov-Smirnov (K-S) tests (ps < 0.001) and Shapiro-Wilk (S-W) tests (ps < 0.001), consistent with the known sensitivity of these tests to minor departures in large samples.

Given that the absolute skewness and kurtosis values fell within the robust ranges for maximum likelihood estimation (|skew| < 3, |kurt| < 10) and considering the central limit theorem’s applicability to large samples, parametric analyses were deemed appropriate for all subsequent tests^55,56^. This decision aligns with methodological recommendations that emphasize practical distributional characteristics over strict statistical significance in large-scale behavioral research (*N* > 200).

Bivariate correlational analyses revealed systematic associations among study variables (Table 1). Key findings included: (1) Significant negative correlations between mindfulness and rumination (*r* = − .20, *p* < .01), alexithymia (*r* = − .25, *p* < .01), and anxiety symptoms (*r* = − .21, *p* < .01); (2) Strong positive associations between rumination and both alexithymia (*r* = .64, *p* < .01) and anxiety symptoms (*r* = .80, *p* < .01); (3) Moderate positive correlation between alexithymia and anxiety symptoms (*r* = .61, *p* < .01). This correlational matrix preliminarily supports rumination and alexithymia as potential mediators in the mindfulness-anxiety pathway.

**Table 1:** Intercorrelations among study Variables.

	M ± SD (*N*)	1	2	3	4
1.Mindfulness	57.22 ± 15.80 (860)	1			
2.Rumination	39.34 ± 12.95 (860)	-0.20^**^	1		
3.Alexithymia	53.29 ± 9.89 (860)	-0.25^**^	0.64^**^	1	
4.Anxiety Symptoms	25.69 ± 17.26 (860)	-0.21^**^	0.80^**^	0.61^**^	1

Note. *N* = 860. Significance levels: ^*^p < .05, ^**^p < .01, ^***^p < .001.

Given moderately strong intervariable correlations (absolute *r* = .20-0.80), we conducted multicollinearity diagnostics to ensure the validity of the hypothesized mediation model. Variance inflation factors (*VIF*) for all predictors ranged from 1.07 to 1.72, indicating no significant multicollinearity. These values substantially undercut both the conventional threshold of 5 recommended for behavioral research and the rigorous 3 criterion proposed in advanced statistical methodologies^57^. Tolerance statistics (0.58–0.94) further corroborated these findings. Collectively, these results confirm the mathematical robustness of parameter estimates, supporting the integrity of subsequent mediation analyses.

Notably, while mindfulness demonstrated a significant bivariate correlation with anxiety symptoms (*r* = − .21, *p* < .01), this association became nonsignificant in multivariate analysis (*β*= − 0.026, *p* = .155). This pattern suggests potential mediation through rumination and alexithymia. To systematically examine these indirect pathways, further serial mediation analyses were warranted.

### Demographic group differences

#### Sex differences

Independent samples *t*-tests revealed significant sex-based differences in alexithymia (*t* = − 2.75, *p* < .01, Cohen’s *d* = 0.27), with males (*M* = 55.53 ± 9.82) scoring higher than females (*M* = 52.91 ± 9.82). No significant sex differences were observed for mindfulness (*t* = 1.86, *p* = .063), rumination (*t* = − 1.56, *p* = .119), or anxiety symptoms (*t* = − 1.46, *p* = .146) (Table 2).

**Table 2:** Sex differences between Variables.

Variables	Sex	M ± SD	t	Cohen’s d
Mindfulness	Male	54.80 ± 16.55	1.86	0.18
	Female	57.64 ± 15.6		
Rumination	Male	41.01 ± 14.08	-1.56	0.15
	Female	39.05 ± 12.73		
Alexithymia	Male	55.53 ± 9.82	-2.75**	0.27
	Female	52.91 ± 9.82		
Anxiety Symptoms	Male	28.02 ± 19.72	-1.46	0.14
	Female	25.90 ± 16.79		

Note. Sex = biological designation at birth. *N* = 860. Significance levels: ^*^p < .05, ^**^p < .01, ^***^p < .001.

#### Grade differences

Significant grade-related variations emerged across all measured constructs (Table 3). One-way ANOVAs demonstrated statistically robust effects for mindfulness (*F*(3, 856) = 11.96, *p* < .001, *η²* = 0.04), rumination (*F*(3, 856) = 12.99, *p* < .001, *η²* = 0.04), alexithymia (*F*(3, 856) = 11.95, *p* < .001, *η²* = 0.04), and anxiety symptoms (*F*(3, 856) = 16.95, *p* < .001, *η²* = 0.06).

**Table 3:** Grade-Level differences in primary Variables.

Variables	Grade	M ± SD	F	η²
Mindfulness	Freshman	60.52 ± 15.90	11.96***	0.04
	Sophomore	53.96 ± 14.30		
	Junior	54.07 ± 16.18		
	Senior	57.46 ± 14.42		
Rumination	Freshman	36.53 ± 11.12	12.999***	0.04
	Sophomore	42.74 ± 13.68		
	Junior	41.05 ± 14.79		
	Senior	40.48 ± 10.50		
Alexithymia	Freshman	51.18 ± 9.53	11.95***	0.04
	Sophomore	54.86 ± 9.31		
	Junior	55.50 ± 10.41		
	Senior	54.78 ± 9.82		
Anxiety Symptoms	Freshman	21.46 ± 14.55	16.95***	0.06
	Sophomore	30.50 ± 18.14		
	Junior	29.04 ± 19.51		
	Senior	25.46 ± 15.79		

Note. *N* = 860. Significance levels: ^*^p < .05, ^**^p < .01, ^***^p < .001.

Bonferroni-corrected post hoc comparisons revealed significant grade-level differences in three psychological constructs after adjusting the alpha level to 0.008 (0.05/6 pairwise comparisons). Freshmen exhibited higher mindfulness than juniors (*ΔM* = 6.46, 95% CI [3.09, 10.03], *p* < .001) and sophomores (*ΔM* = 6.56, [2.91, 10.01], *p* < .001). Sophomores reported greater rumination (*ΔM* = 6.21, [3.37, 9.05], *p* < .001) and anxiety (*ΔM* = 5.04, [1.23, 8.85], *p* = .006) compared to seniors. No significant differences emerged for alexithymia across grades (all *p* > .008). These findings highlight distinct developmental trajectories in mental health indicators during college years.

### Serial mediation analysis

A serial mediation model was tested using Hayes’ PROCESS macro (Model 6), with mindfulness as the predictor, anxiety symptoms as the outcome, and rumination/alexithymia as mediators, controlling for sex and academic grade. Bootstrap resampling (5,000 iterations) with bias-corrected 95% confidence intervals (CIs) was employed, where effects were deemed significant if CIs excluded zero.

As presented in Table 4, mindfulness demonstrated a statistically significant direct negative association with anxiety symptoms before accounting for mediators (*β* = -0.182, *t* = -5.44, *p* < .001), confirming Hypothesis 1. Subsequent mediation analysis revealed full mediation effects, as the direct pathway became nonsignificant upon introducing mediators (*β* = -0.026, *t* = -1.24, *p* = .216). Mindfulness showed significant negative associations with both rumination (*β* = -0.176, *t* = -5.23, *p* < .001) and alexithymia (*β* = -0.119, *t* = -4.48, *p* < .001). Furthermore, rumination demonstrated strong positive associations with alexithymia (*β* = 0.599, *t* = 22.49, *p* < .001) and anxiety symptoms (*β* = 0.695, *t* = 26.89, *p* < .001), while alexithymia maintained an independent positive association with anxiety symptoms (*β* = 0.152, *t* = 5.79, *p* < .001).

Covariate analysis identified male participants as having significantly higher levels of alexithymia (*β* = 0.051, *t* = 1.98, *p* < .05). Notably, academic progression was associated with lower rumination (*β* = -0.121, *t* = -3.60, *p* < .001) and alexithymia (*β* = -0.068, *t* = -2.56, *p* < .05).

**Table 4:** Regression coefficients for sequential mediation Model.

Regression Equation		Fitting index			Significance		Confidence Interval	
Result variable	Predictor variable	*R*	*R²*	*F*	*β*	*t*	LLCI	ULCI
Anxiety Symptoms	Mindfulness	0.250	0.063	19.06^***^	-0.182	-5.44^***^	-0.271	-0.127
	Sex				0.039	1.18	-1.269	-5.104
	Grade				-0.138	-4.11^***^	-3.708	-1.313
Rumination	Mindfulness	0.234	0.055	16.49^***^	-0.176	-5.23^***^	-0.198	-0.090
	Sex				0.038	1.13	-1.021	-0.090
	Grade				-0.121	-3.60^***^	-2.558	-0.753
Alexithymia	Mindfulness	0.653	0.427	159.26^***^	-0.119	-4.48^***^	-0.107	-0.042
	Rumination				0.599	22.49^***^	0.418	0.497
	Sex				0.051	1.98^*^	0.011	2.870
	Grade				-0.068	-2.56^*^	-1.248	-0.166
Anxiety Symptoms	Mindfulness	0.813	0.661	332.71^***^	-0.026	-1.24	-0.073	0.016
	Rumination				0.695	26.89^***^	0.859	0.995
	Alexithymia				0.152	5.79^***^	0.176	0.356
	Sex				0.002	0.09	-1.836	2.014
	Grade				-0.032	-1.58	-1.316	0.143

Note. *N* = 860. All coefficients represent standardized estimates. Significance levels: ^*^p < .05, ^**^p < .01, ^***^p < .001.

**Fig. 2:**
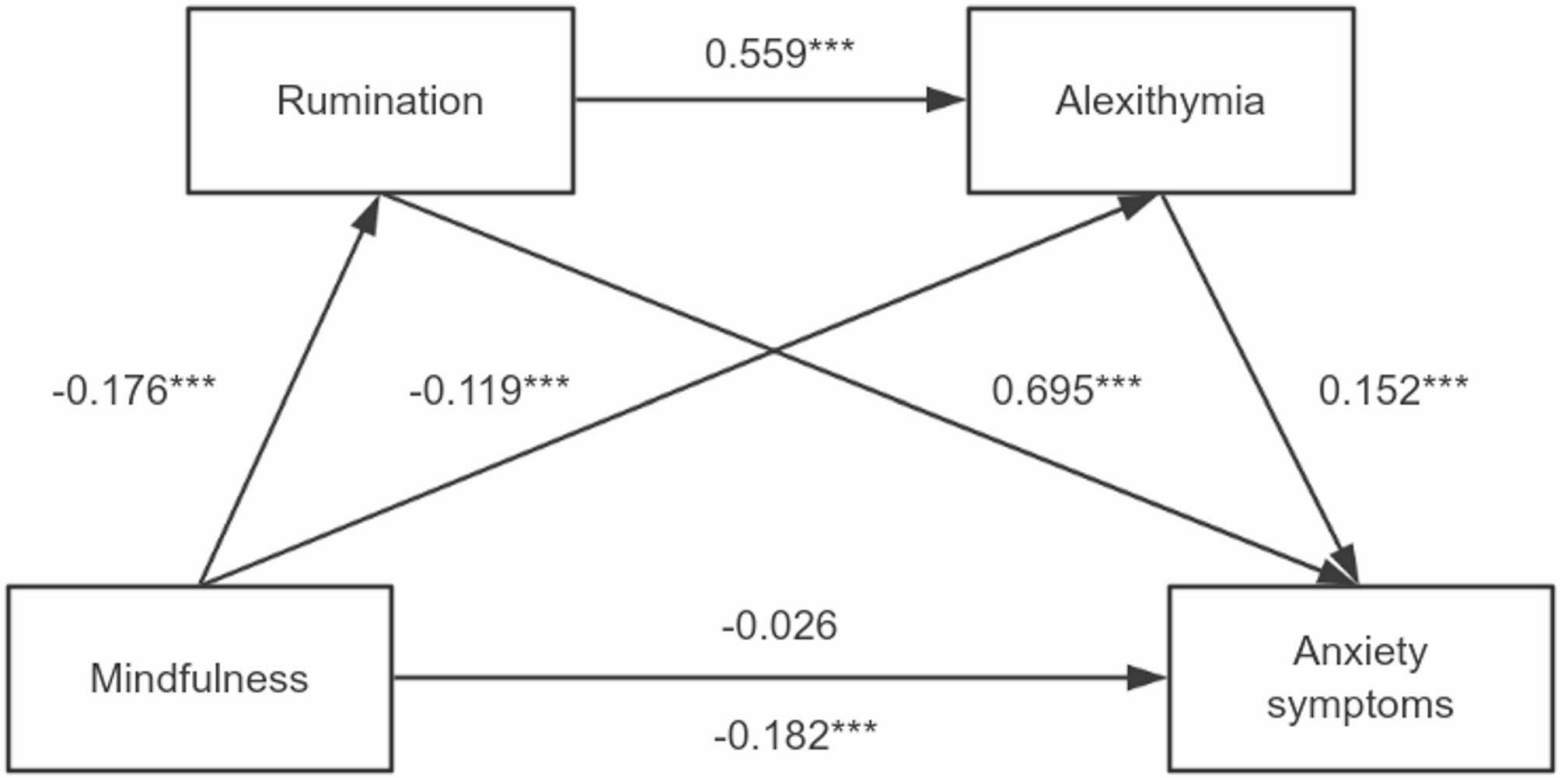
Serial Mediation Pathways Note. *N* = 860. Standardized regression coefficients derived from PROCESS Model 6. Solid paths indicate significant mediation effects (^***^p < .001). The model demonstrates serial mediation through rumination and alexithymia in the mindfulness-anxiety relationship.

Bootstrap mediation analysis with bias-corrected percentile method revealed significant indirect pathways between mindfulness and anxiety symptoms (Table 5). The total indirect effect reached − 0.157 (95% CI [-0.224, -0.090]), accounting for 86.26% of the total effect, indicating predominant mediation through specified pathways. Specifically, the indirect effect through rumination alone (Ind1: M→Rum→AS) was − 0.122 (95% CI [-0.181, -0.065]), explaining 67.03% of the total effect and supporting Hypothesis H2. The independent mediation through alexithymia (Ind2: M→Alex→AS) showed a smaller but significant effect of -0.018 (95% CI [-0.029, -0.009]), representing 9.89% of the total effect and confirming Hypothesis H3. Notably, the serial mediation through rumination→alexithymia (Ind3: M→Rum→Alex→AS) demonstrated a significant serial indirect effect of -0.016 (95% CI [-0.026, -0.008]), accounting for 8.79% of the total effect and validating Hypothesis H4. These findings point to rumination and alexithymia operating as sequential mediators, with rumination demonstrating the strongest mediating role (Fig. 2).

Contrast analysis revealed significant differences in mediation pathway strengths: The rumination pathway (Ind1) demonstrated substantially greater mediation than the alexithymia pathway (Ind1 vs. Ind2: C1 = -0.104, 95% CI [-0.161, -0.049]), highlighting the stronger mediating role of rumination. Furthermore, the direct rumination pathway (Ind1) showed significantly larger effects than the sequential rumination→alexithymia chain (Ind1 vs. Ind3: C2 = -0.106, 95% CI [-0.158, -0.056]), underscoring the primacy of rumination’s direct influence. Importantly, no significant difference emerged between the independent alexithymia pathway and the sequential chain (Ind2 vs. Ind3: C3 = -0.002, 95% CI [-0.012, 0.007]), suggesting comparable effect sizes between these two mediation routes. These contrasts collectively reinforce rumination’s centrality while delineating distinct mechanistic contributions of the proposed pathways.

**Table 5:** Decomposition of Mindfulness-Anxiety mediation Effects.

Effect Type	Effect size	Boot SE	95% CI	Proportion (%)
Total Indirect Effect	-0.157	0.034	[-0.224,-0.090]	86.26
Ind1: M→Rum→AS	-0.122	0.029	[-0.181,-0.065]	67.03
Ind2: M→Alex→AS	-0.018	0.005	[-0.029,-0.009]	9.89
Ind3: M→Rum→Alex→AS	-0.016	0.005	[-0.026,-0.008]	8.79
C1	-0.104	0.029	[-0.161,-0.049]	–
C2	-0.106	0.026	[-0.158,-0.056]	–
C3	-0.002	0.005	[-0.012,0.007]	–

Note. *N* = 860. All variables standardized. M = Mindfulness; Rum = Rumination; Alex = Alexithymia; AS = Anxiety Symptoms; *Boot SE* = Bootstrap standard error; 95% CI = Bias-corrected confidence interval. Proportion calculation: (Indirect effect / Total effect) × 100, where Total effect = -0.182. Contrast definitions: C1 = Ind1 - Ind2; C2 = Ind1 - Ind3; C3 = Ind2 - Ind3.

### Comparisons of competing mediation sequences

To address theoretical concerns regarding the directionality of the rumination-alexithymia relationship, supplementary analyses compared three competing mediation sequences using PROCESS Macro Models 4 and 6. These analyses assessed the robustness and directionality of the proposed mediation structure:

Model A (Original sequence): mindfulness → rumination → alexithymia → anxiety.

Model B (Reversed sequence): mindfulness → alexithymia → rumination → anxiety.

Model C (Parallel mediators): mindfulness → [rumination + alexithymia] → anxiety.

All models controlled for sex and grade covariates and employed 5,000 bootstrap samples for bias-corrected confidence intervals. Model adequacy was evaluated against four criteria: explained variance (*R²*), magnitude and significance of indirect effects, statistical significance of individual path coefficients, and model parsimony (Supplementary Tables S2-S5; Supplementary Figs. S1-S2).

Firstly, all three models explained comparable variance in anxiety symptoms (R² = 0.661). However, rumination accounted for 42.7% of the variance in alexithymia within Model A, whereas alexithymia explained only 40.6% of the variance in rumination within Model B. This indicates stronger predictive validity for the pathway from rumination to alexithymia.

Subsequently, in Model A, the serial indirect effect (mindfulness → rumination → alexithymia → anxiety) was significant (β = -0.016, 95% CI [-0.026, -0.008]), accounting for 8.79% of the total indirect effect. Model B’s reversed chain mediation (mindfulness → alexithymia → rumination → anxiety) accounted for 53.27% of the indirect effect; however, its specific indirect path through rumination alone (mindfulness → rumination → anxiety) was non-significant. Model C (parallel mediation) confirmed rumination as the dominant mediator (67.34% of the total indirect effect), followed by alexithymia (18.59%).

Thirdly, Model A demonstrated statistically significant coefficients for all hypothesized paths: Mindfulness → Rumination (β = -0.176, *p* < .001); Rumination → Alexithymia (β = 0.599, *p* < .001); Alexithymia → Anxiety (β = 0.152, *p* < .001). In contrast, Model B exhibited a non-significant path from mindfulness to rumination (β = -0.030, *p* > .05), which compromised the mediation premise of this model.

Finally, while Model C demonstrates greater parsimony, it lacks explanatory nuance regarding the potential sequential relationship between mediators. Model A provides superior theoretical justification and empirical coherence.

Collectively, the theoretical rationale and empirical findings support the hypothesized chain mediation pathway: mindfulness → rumination → alexithymia → anxiety. This sequence demonstrates statistical robustness, theoretical plausibility, and greater consistency than alternative models. Nonetheless, the cross-sectional design precludes causal inference. As Hayes emphasized, testing competing mediation models represents a valid strategy for examining directional assumptions in the absence of longitudinal data^58^.

## Discussion

The current study examined the association between mindfulness and anxiety symptoms in a predominantly female (85%) and freshman-skewed (46.5%) sample of Chinese university students, revealing rumination and alexithymia as serial mediators. These findings support the inverse association between mindfulness and anxiety symptoms within this specific cohort and demonstrate its links through cognitive-emotional pathways. This evidence offers preliminary insights into mindfulness-anxiety relationships in similar demographic contexts, though generalizability to broader student populations requires verification.

### The direct relationship between mindfulness and anxiety symptoms

Consistent with prior evidence, our results confirmed a significant negative correlation between mindfulness and anxiety symptom severity^59,60^. This correlation suggests that heightened present-moment awareness and reduced hyperreactivity to adverse stimuli are characteristic features co-occurring with lower anxiety levels.

However, the nonsignificant direct effect after mediator inclusion implies that the observed association between mindfulness and anxiety symptoms may be predominantly accounted for by their shared associations with cognitive and emotional regulatory processes, rather than implying direct pathways. This pattern aligns with the Mindfulness Reperceiving Model, which theorizes decentering as a metacognitive state characterized by disengagement from maladaptive mental content alongside objective experiential observation^61^. Within this theoretical model, mindfulness conceptually co-occurs with modified cognitive appraisals of negative stimuli (e.g., lower rumination levels) and greater emotional adaptability (e.g., lower alexithymia)^62,63^. The observed attenuation of sustained activation in anxiety-related neural circuits is conceptually parallel to this dual regulatory pattern, potentially accounting for the observed mediation relationship and highlighting mindfulness’s systemic theoretically proposed therapeutic correlates^64^.

Furthermore, mindfulness demonstrates robust correlations with anxiety symptom in academic populations, conceivably mediated by enhanced self-regulatory capacities and emotional attunement^64,65^. Empirical evidence demonstrates that mindfulness-based interventions (MBIs) effectively mitigate anxiety symptoms in university students, with mindfulness training correlating with improved stress coping and reduced emotional avoidance^66–69^. Theoretically, by fostering metacognitive regulation and attenuating maladaptive avoidance patterns, mindfulness may strengthen emotion regulation competencies conceptually critical for anxiety reduction^70^. Collectively, these findings position the potential of mindfulness enhancement as a strategy worthy of investigation for addressing anxiety in academically pressured student populations.

### The mediating role of rumination

This study identified rumination as a pivotal mediator in the observed association between mindfulness and anxiety, reinforcing its status as a core cognitive vulnerability factor for anxiety pathology^71^. The Self-Regulatory Executive Function Model conceptualizes rumination as a maladaptive metacognitive process theoretically implicated in amplifying negative emotional states through sustained cognitive fixation on distress^72^. Functionally associated with ineffective emotion regulation, rumination is linked to exacerbation of anxiety through prolonging negative affectivity and heightening threat vigilance^73,74^. Neurobiological evidence links rumination to hyperactivation of the Default Mode Network (DMN) during self-referential processing and impaired inhibitory control mediated by Frontoparietal Network (FPN) dysregulation^75^. Our findings substantiate rumination’s mediating role in the observed mindfulness-anxiety association, consistent with theories describing a pathogenic “anxiety-rumination-anxiety escalation” cycle^74,76^. Mindfulness may disrupt this theoretically proposed maladaptive loop through enhanced cognitive flexibility and attentional disengagement from negative ideation, aligning with the Mindful Emotion Regulation Model’s postulates^77^.

Elevated mindfulness levels are correlated with attenuated rumination severity, which in turn are associated with reduced anxiety symptoms. This underscores the correlative pattern where higher mindfulness co-occurs with restructured maladaptive cognitive schemas^78,79^. Integrative interventions simultaneously targeting mindfulness enhancement and rumination reduction are plausible and worthy of investigation for optimizing therapeutic outcomes for anxiety symptoms^78^. Systematic mindfulness training could potentially mitigate rumination’s frequency and emotional intensity, thereby potentially contributing to alleviated anxiety burden in student populations.

### The mediating role of alexithymia

Alexithymia emerged as a significant independent mediator, suggesting an association wherein higher mindfulness co-occurs with lower anxiety symptoms partly through its correlation with improved emotional differentiation and expression. This construct demonstrates robust predictive validity for anxiety symptom severity, particularly in correlation with deficient interoceptive awareness—the impaired capacity to detect and interpret somatic-affective signals^80–82^.

Our results align with evidence showing an association between mindfulness training and reduced alexithymic traits, conceivably mediated by enhanced self-concept coherence and emotional granularity^83,84^. Neuroplasticity research reveals correlative evidence that mindfulness strengthens functional connectivity between interoceptive hubs (anterior insula) and emotional regulation centers (dorsolateral prefrontal cortex), which theoretically could facilitate the neural encoding of visceral states into conscious emotional experiences^85^. This neurocognitive remodeling may be a potential neural substrate underlying mindfulness-induced improvements in affective labeling accuracy—a core deficit in alexithymia.

The observed mediation pattern corresponds with theoretical frameworks describing dual correlative pathways: (1) greater meta-awareness of transient emotional states associated with decentering, and (2) lower experiential avoidance associated with emotional disengagement^86^. Academically stressed students exhibiting greater emotional literacy show associations with enhanced differentiation between normative stress responses (e.g., exam-related arousal) and pathological anxiety, concomitant with attenuated catastrophizing interpretations of physiological activation^84^.

Clinically, mindfulness protocols incorporating alexithymia-focused modules are theoretically grounded and warrant investigation for optimizing outcomes for patients with somatized psychological distress^82^. The persistent mediation effect after controlling for rumination confirms alexithymia’s distinct correlative affective pathway, suggesting potential value in dual-focused interventions addressing both cognitive (rumination) and emotional (alexithymia) processing deficits.

### Potential theoretical pathways of serial associations​

This study observed a significant sequential statistical pattern linking mindfulness to anxiety symptoms via rumination and alexithymia in this specific association analysis, highlighting cognitive-emotional interconnections relevant to understanding mindfulness-based approaches in theoretical models. Based on the observed correlation pattern and theory, one speculative sequential model could involve: Higher levels of chronic rumination are correlated with reduced executive resource availability and attenuated metacognitive monitoring capacities, which in turn are associated with the emotional disorientation and expressive limitations characteristic of alexithymia^74,87^. This observed progressive statistical association could theoretically reflect (but does not prove) a sequence where compromised emotional identification is associated with intensified physiological hyperarousal, which is subsequently linked to catastrophizing interpretations of somatic signals^88^.

Neuroimaging evidence provides correlative support: Mindfulness meditation training (MMT) is associated with modulated functional interactions between the default mode network (DMN, linked to rumination) and the frontoparietal network (FPN, involved in cognitive control)^75^. Functional MRI data reveal MMT-induced correlated hyperactivation in brain regions critical for attentional deployment and emotional reappraisal (e.g., superior frontal gyrus, posterior cingulate cortex, right hippocampus)^85^. These neuroplastic adaptations are conceptually consistent with enhanced DMN-FPN decoupling, theoretically disrupting rumination’s neural substrates while potentially strengthening interoceptive awareness.

The observed serial mediation pathway (mindfulness → rumination → alexithymia → anxiety), accounting for 8.79% of the total indirect effect, highlights interconnected cognitive-affective mechanisms underlying mindfulness-anxiety relationships. While rumination demonstrated substantial mediation (67.03% of total indirect effects), we acknowledge potential conceptual overlap between rumination measures (e.g., RRS symptom-focused items like “I think about how sad I feel”) and anxiety symptomatology assessed through SCAARED (e.g., persistent worry items). This measurement limitation necessitates careful interpretation of rumination’s process-based role, particularly given content similarities that might inflate statistical associations.

Three lines of evidence from our study support rumination’s distinct mechanistic contribution beyond measurement artifacts. First, consistent with the Self-Regulatory Executive Function (S-REF) model, rumination operates as a preceding cognitive process characterized by persistent threat monitoring22, which theoretically amplifies anxiety vulnerability through neurobiologically distinct pathways - default mode network hyperactivity for rumination versus limbic system dysregulation for anxiety75. Second, our competing mediation models (Supplementary Figs. S1) empirically validated rumination’s temporal precedence, as reversing the mediator sequence (mindfulness → alexithymia → rumination → anxiety) rendered the mindfulness→rumination path nonsignificant (β = -0.030, *p* > .05). Third, when modeled concurrently (Table 4), rumination maintained robust predictive validity for anxiety (β = 0.695, *p* < .001) despite nonsignificant direct mindfulness effects (β = -0.026, *p* = .216), indicating its unique explanatory power. As visually reinforced in Fig. 2, rumination’s dual predictive relationships - significantly influencing alexithymia (β = 0.599) while independently contributing to anxiety (β = 0.152) - substantiate its process-oriented function beyond symptom covariance.

Collectively, while measurement limitations warrant consideration, our integrated theoretical framework and empirical findings affirm rumination’s role as a primary causal antecedent rather than mere symptom correlate. Future investigations employing ecological momentary assessment could further temporally disentangle state rumination from anxiety manifestations.

### Integrated pathway patterns and research implications

This study revealed specific patterns of statistical association. The sequential cognitive-affective pathway (mindfulness → rumination → alexithymia → anxiety symptoms) was statistically supported in our correlation model, and we quantified the proportional variance explained by different paths. Three key association patterns emerge:

The proportional strength of associations differs substantially across pathways. Rumination demonstrated the strongest linkage within the overall indirect association pattern, accounting for 67.03% of the total statistical indirect effect. This substantially exceeds both the independent association through alexithymia (9.89%) and the sequential path through rumination to alexithymia (8.79%). This quantification provides new perspective on the relative weight of cognitive versus affective processes in this population.

Among theoretically plausible sequences, the pathway from mindfulness through rumination to alexithymia to anxiety showed complete statistical support within this model: all component paths were significant, the specific indirect effect for the sequence was robust (β= -0.016, 95% CI [-0.026, -0.008]), and alternative sequences demonstrated contradictory statistical evidence. This pattern aligns with conceptual models suggesting cognitive processes may influence subsequent emotional processing.

These statistical patterns—where rumination exhibits the strongest proportional association and the initial position in the statistically best-supported sequence—provide a foundation for generating hypotheses. This pattern suggests the potential value of investigating rumination reduction as a target that might simultaneously correlate strongly with the primary pathway associated with anxiety symptoms and be linked to subsequent emotional awareness difficulties. This represents a testable model for future longitudinal or experimental research seeking to understand temporal dynamics and intervention efficiency.​.

Collectively, these patterns provide a more nuanced understanding of how cognitive and affective processes are statistically interrelated within the observed mindfulness-anxiety association in this study. We explicitly emphasize that the cross-sectional design cannot establish temporal sequencing or causality between these factors; verification of such sequences requires prospective or experimental designs.​.

### Exploratory clinical framework

Based only on the observed cross-sectional serial mediation patterns, we propose a conjectural conceptual framework outlining three potentially sequential, but statistically indistinguishable, components in the observed association pattern: cognitive disengagement, emotional clarification, and symptom mitigation. This model is explicitly presented as a theoretically plausible interpretation derived from both the statistical associations and established theoretical mindfulness-anxiety pathways^70,72,75,77^. We emphasize that it reflects conceptual linkage within the observed correlations and is NOT an empirically validated causal sequence or mechanism derived from this study’s design.​.

Initially, the observed correlation between mindfulness training and reduced rumination could theoretically reflect the engagement of metacognitive awareness and decentering capacities, core mechanisms within mindfulness-anxiety research^75^. Theoretically, such shifts might involve modulation of Default Mode Network (DMN) activity^72^.

Subsequently, the observed correlations show reductions in alexithymic tendencies associated with features related to emotional granularity. Mindfulness-anxiety pathway research theorizes this may relate to enhanced interoceptive awareness^70^.

Ultimately, the observed correlations involve reductions in anxiety symptoms, conceptually consistent with integrated therapeutic outcomes described theoretically in mindfulness-anxiety pathway models^77^.

Clinically, this conjectural multi-phase association pattern could translate into a structured theoretical clinical protocol framework emphasizing three core elements: Target, Focus, and Reinforcement. The Target stage could theoretically prioritize cognitive restructuring aimed at countering rumination, utilizing mindfulness to potentially foster metacognitive detachment. The Focus stage could theoretically transition to emotion differentiation training, employing body-mind integration exercises to potentially enhance precision in affective labeling. The Reinforcement stage could theoretically consolidate gains through psychoeducation on normative stress physiology, aiming at decoupling residual physiological arousal from catastrophic interpretations^71^. This hierarchical clinical model embodies similar conceptual principles—from cognitive disengagement to emotional refinement—foundational to theoretical mindfulness-anxiety pathways, proposing an integrated approach whose efficacy and causal sequence require rigorous empirical validation.​.

### Limitations and future directions

The current study has several limitations requiring careful consideration. First, the present study utilized a binary biological sex classification (male/female) derived from birth-assigned status, with no assessment of gender identity or sociocultural variables. Consequently, the findings may lack generalizability to non-binary, intersex, or transgender populations. Future investigations should integrate multidimensional gender constructs—such as self-identified gender identity, social roles, and hormonal profiles—to improve inclusivity and analytical validity.

The demographic composition of our sample represents a significant methodological consideration. With female participants comprising 85.5% of respondents and freshmen constituting nearly half (46.5%) of the cohort—while seniors were notably underrepresented at 5.3%—these imbalances introduce important constraints on the generalizability of our findings. This skewed distribution may have amplified observed anxiety prevalence rates (49.19% meeting clinical thresholds), particularly given established gender differences in emotional symptom reporting patterns where females demonstrate heightened vulnerability to internalizing disorders. The freshman predominance further limits our ability to map developmental trajectories across academic stages, as critical transitions like the sophomore-year anxiety surge observed in Table 3 require more balanced representation. While observed sex differences in alexithymia are consistent with prior evidence, recent meta-analyses suggest these disparities may be relatively modest^89–91^. The freshman predominance may have influenced developmental pattern observations, particularly the peak anxiety and rumination levels among sophomores—a finding diverging from certain existing studies^92,93^. These sampling characteristics likely interact with China’s unique academic pressures—including residual Gaokao examination stress and career competition anxieties—creating a context-specific profile that warrants verification in more heterogeneous cohorts. Future investigations should therefore implement stratified sampling frameworks ensuring proportional gender and academic-year representation, while incorporating comprehensive gender identity measures to disentangle biological versus sociocultural influences on anxiety pathways. Longitudinal tracking across critical transition periods (e.g., freshman to sophomore year) would further elucidate developmental patterns suggested but not confirmed by our cross-sectional grade comparisons.

The cross-sectional nature of this study fundamentally limits any causal interpretations of the serial mediation effects. While our model statistically links mindfulness and anxiety symptoms through mediators, any directional interpretation (e.g., mindfulness influencing mediators, which then influence anxiety) remains speculative based on this design. Reverse or reciprocal causation are equally plausible alternative explanations for the observed association patterns. Future research must implement longitudinal designs incorporating mindfulness-based interventions, coupled with experimental manipulation of mediators, to empirically investigate temporal precedence and potential causal hierarchy.​.

Methodological limitations related to self-report measures introduce potential response biases, including social desirability effects and common method variance. Future research would benefit from multimodal assessment approaches, such as behavioral paradigms (e.g., emotional granularity tasks for alexithymia), ecological momentary assessment of rumination patterns, and physiological anxiety biomarkers (e.g., cortisol levels, heart rate variability).

Finally, the cultural specificity of findings necessitates systematic investigation. China’s competitive academic environment and collectivist social norms may foster distinct manifestations of mindfulness and anxiety symptoms compared to Western contexts. Cross-cultural replications using equivalent methodologies could disentangle universal versus culture-bound aspects of these relationships, particularly concerning emotion regulation strategies^94,95^. To advance theoretical understanding and clinical applications, we propose three research priorities: (1) multi-wave longitudinal studies mapping developmental trajectories across university transitions; (2) mechanism-focused randomized controlled trials of mindfulness interventions; and (3) cross-national comparative research informing culturally adapted mental health strategies.

## Conclusion

This study reveals a pattern of statistical associations linking mindfulness with lower anxiety symptom levels in Chinese university students demographically aligned with our sample (predominantly female and early-stage undergraduates). The serial mediation model demonstrates a pattern of covariation where mindfulness is correlated with lower anxiety symptom severity, and this overall correlation is statistically accounted for, in significant part, by its covariation with less frequent rumination patterns and lower alexithymia. This mediation pattern fits neurocognitive frameworks describing correlations between mindfulness practice, attenuated default mode network hyperactivity, and heightened interoceptive awareness.

For populations sharing our sample’s characteristics, the results indicate potential associations supporting investigation into mindfulness-based approaches addressing negative thinking and emotion differentiation capacities during initial university years. Critical sample constraints—specifically gender imbalance (85% female) and freshman overrepresentation (46.5%)—restrict broader generalizability.

Future longitudinal research employing representative sampling is essential to investigate the temporal dynamics and potential causal relationships underlying these associations across diverse academic populations.

## Electronic supplementary material

Below is the link to the electronic supplementary material.

Supplementary Material 1

## Data Availability

The datasets generated during and/or analysed during the current study are available from the corresponding author on reasonable request. The data are not publicly available due to privacy and ethical restrictions related to participant confidentiality. Supplementary materials referenced in this study are permanently archived through the journal’s Electronic Supplementary Materials system, accessible via the article’s online version.
