# Supplementary material for: Rumination and alexithymia serially mediate the relationship between mindfulness and anxiety symptoms in Chinese university students
Source: Sci Rep. 2025 Jul 16;15:25697. doi: 10.1038/s41598-025-11973-0 (PMC12267692; doi:10.1038/s41598-025-11973-0)
Supplement: Supplementary file 1 — Supplementary Material 1 [file 41598_2025_11973_MOESM1_ESM.docx]

| Variable | Skewness(SE) | Kurtosis(SE) | Shapiro-Wilk | Kolmogorov-Smirnov |
| --- | --- | --- | --- | --- |
| Mindfulness | -0.357(0.083) | 0.432(0.167) | 0.976^***^ | 0.087^***^ |
| Rumination | 1.037(0.083) | 1.666(0.167) | 0.923^***^ | 0.122^***^ |
| Alexithymia | 0.021(0.083) | -0.322(0.167) | 0.985^***^ | 0.087^***^ |
| Anxiety Symptoms | 0.774(0.083) | 0.425(0.167) | 0.934^***^ | 0.094^***^ |

**Supplementary Table S1.** Kurtosis, Skewness, and Normality Test

Note. *N* = 860. Significance levels: ^*^*p* < .05, ^**^*p* < .01, ^***^*p* < .001.

**Supplementary Table S2.** Regression Coefficients for Sequential Mediation Model

| Regression Equation | |  | Fitting index | | |  | Significance | |  | Confidence Interval | |
| --- | --- | --- | --- | --- | --- | --- | --- | --- | --- | --- | --- |
| Result variable | Predictor variable |  | *R* | *R²* | *F* |  | *β* | *t* |  | LLCI | ULCI |
| Anxiety Symptoms | Mindfulness |  | 0.250 | 0.063 | 19.06^***^ |  | -0.199 | -5.44^***^ |  | -0.271 | -0.127 |
|  | Sex |  |  |  |  |  | 1.918 | 1.18 |  | -1.269 | 5.104 |
|  | Grade |  |  |  |  |  | -2.511 | -4.11^***^ |  | -3.709 | -1.313 |
| Alexithymia | Mindfulness |  | 0.297 | 0.088 | 27.50^***^ |  | -0.141 | -6.79^***^ |  | -0.181 | -0.100 |
|  | Sex |  |  |  |  |  | 2.071 | 2.26^*^ |  | 0.270 | 3.872 |
|  | Grade |  |  |  |  |  | -1.464 | -4.25^***^ |  | -2.141 | -0.787 |
| Rumination | Mindfulness |  | 0.637 | 0.406 | 146.13^***^ |  | -0.030 | -1.33 |  | -0.074 | 0.014 |
|  | Alexithymia |  |  |  |  |  | 0.813 | 22.49^***^ |  | 0.742 | 0.883 |
|  | Sex |  |  |  |  |  | -0.304 | -0.31 |  | -2.213 | 1.605 |
|  | Grade |  |  |  |  |  | -0.466 | -1.27 |  | -1.189 | 0.257 |
| Anxiety Symptoms | Mindfulness |  | 0.813 | 0.661 | 332.71^***^ |  | -0.028 | -1.24 |  | -0.073 | 0.016 |
|  | Alexithymia |  |  |  |  |  | 0.266 | 5.79^***^ |  | 0.176 | 0.356 |
|  | Rumination |  |  |  |  |  | 0.927 | 26.89^***^ |  | 0.859 | 0.995 |
|  | Sex |  |  |  |  |  | 0.089 | 0.09 |  | -1.836 | 2.014 |
|  | Grade |  |  |  |  |  | -0.587 | -1.58 |  | -1.316 | 0.143 |

Note. *N* = 860. All coefficients represent standardized estimates. Significance levels: ^*^*p* < .05, ^**^*p* < .01, ^***^*p* < .001.

**Supplementary Table S3.** Decomposition of Mindfulness-Anxiety Mediation Effects

| Effect Type | Effect size | *Boot SE* | 95% CI | Proportion (%) |
| --- | --- | --- | --- | --- |
| Total Indirect Effect | -0.171 | 0.040 | [ -0.250,-0.094] | 85.93 |
| Ind1: M→Alex→AS | -0.037 | 0.010 | [ -0.059,-0.020] | 18.59 |
| Ind2: M→Rum→AS | -0.028 | 0.021 | [-0.0684,0.012] | 14.07 |
| Ind3: M→Alex→Rum→AS | -0.106 | 0.022 | [-0.150,-0.065] | 53.27 |
| C1 | -0.010 | 0.021 | [-0.050,0.32] | – |
| C2 | 0.069 | 0.017 | [0.039,0.105] | – |
| C3 | 0.078 | 0.026 | [-0.028,0.132] | – |

Note. *N* = 860. All variables standardized. M = Mindfulness; Rum = Rumination; Alex = Alexithymia; AS = Anxiety Symptoms; *Boot SE* = Bootstrap standard error; 95% CI = Bias-corrected confidence interval. Proportion calculation: (Indirect effect / Total effect) × 100, where Total effect = -0.199. Contrast definitions: C1 = Ind1 - Ind2; C2 = Ind1 - Ind3; C3 = Ind2 - Ind3.

**Supplementary Table S4.** Regression Coefficients for Parallell Mediation Model

| Regression Equation | |  | Fitting index | | |  | Significance | |  | Confidence Interval | |
| --- | --- | --- | --- | --- | --- | --- | --- | --- | --- | --- | --- |
| Result variable | Predictor variable |  | *R* | *R²* | *F* |  | *β* | *t* |  | LLCI | ULCI |
| Anxiety Symptoms | Mindfulness |  | 0.250 | 0.063 | 19.06^***^ |  | -0.199 | -5.44^***^ |  | -0.271 | -0.127 |
|  | Sex |  |  |  |  |  | 1.918 | 1.18 |  | -1.269 | 5.104 |
|  | Grade |  |  |  |  |  | -2.511 | -4.11^***^ |  | -3.709 | -1.313 |
| Rumination | Mindfulness |  | 0.234 | 0.055 | 16.49^***^ |  | -0.144 | -5.23^***^ |  | -.198 | -0.090 |
|  | Sex |  |  |  |  |  | 1.379 | 1.13 |  | -1.021 | 3.779 |
|  | Grade |  |  |  |  |  | -1.656 | -3.60^***^ |  | -2.556 | -0.753 |
| Alexithymia | Mindfulness |  | 0.297 | 0.088 | 27.50^***^ |  | -0.141 | -1.46^***^ |  | -0.181 | -0.100 |
|  | Sex |  |  |  |  |  | 2.071 | 2.26^*^ |  | 0.270 | 3.872 |
|  | Grade |  |  |  |  |  | -1.464 | -4.25^***^ |  | -2.141 | -0.787 |
| Anxiety Symptoms | Mindfulness |  | 0.813 | 0.661 | 332.71^***^ |  | -0.028 | -1.24 |  | -0.073 | 0.016 |
|  | Rumination |  |  |  |  |  | 0.927 | 26.89^***^ |  | 0.856 | 0.995 |
|  | Alexithymia |  |  |  |  |  | 0.266 | 5.79^***^ |  | 0.176 | 0.356 |
|  | Sex |  |  |  |  |  | 0.089 | 0.09 |  | -1.836 | 2.014 |
|  | Grade |  |  |  |  |  | -0.587 | -1.58 |  | -1.316 | 0.143 |

Note. *N* = 860. All coefficients represent standardized estimates. Significance levels: ^*^*p* < .05, ^**^*p* < .01, ^***^*p* < .001.

**Supplementary Table S5.** Decomposition of Mindfulness-Anxiety Mediation Effects

| Effect Type | Effect size | *Boot SE* | 95% CI | Proportion (%) |
| --- | --- | --- | --- | --- |
| Total Indirect Effect | -0.171 | 0.040 | [ -0.250,-0.094] | 85.93 |
| Ind1: M→Rum→AS | -0.134 | 0.033 | [ -0.200,-0.069] | 67.34 |
| Ind2: M→Alex→AS | -0.037 | 0.010 | [-0.058 ,-0.021] | 18.59 |
| C1 | -0.096 | 0.029 | [-0.1548,-0.042] | – |

Note. *N* = 860. All variables standardized. M = Mindfulness; Rum = Rumination; Alex = Alexithymia; AS = Anxiety Symptoms; *Boot SE* = Bootstrap standard error; 95% CI = Bias-corrected confidence interval. Proportion calculation: (Indirect effect / Total effect) × 100, where Total effect = -0.199. Contrast definitions: C1 = Ind1 - Ind2.


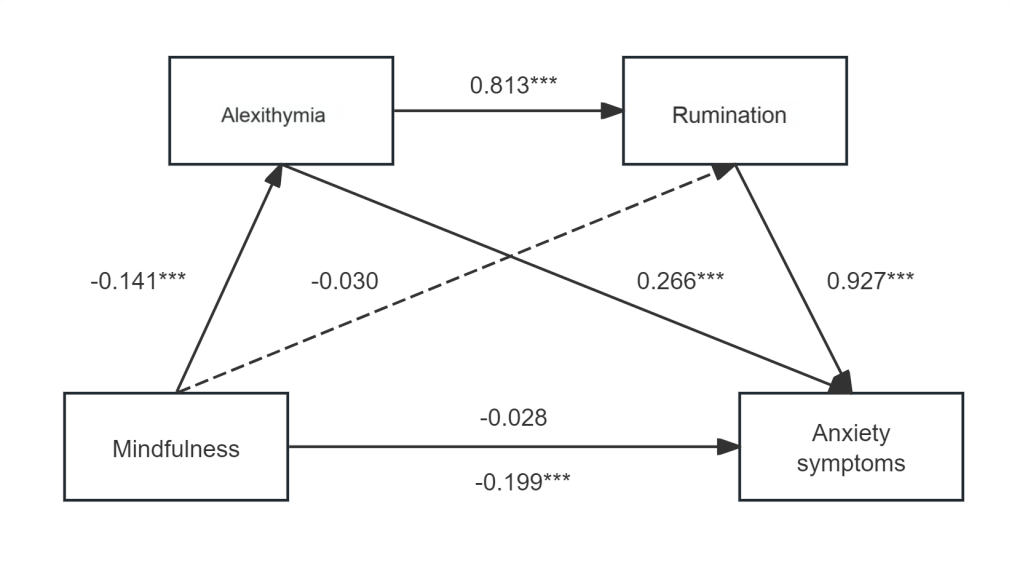


**Supplementary Fig. S1.** Serial Mediation Pathways

Note. N = 860. Standardized regression coefficients derived from PROCESS Model 6. Solid paths indicate significant mediation effects (***p < .001). The model demonstrates serial mediation through alexithymia and rumination in the mindfulness-anxiety relationship.


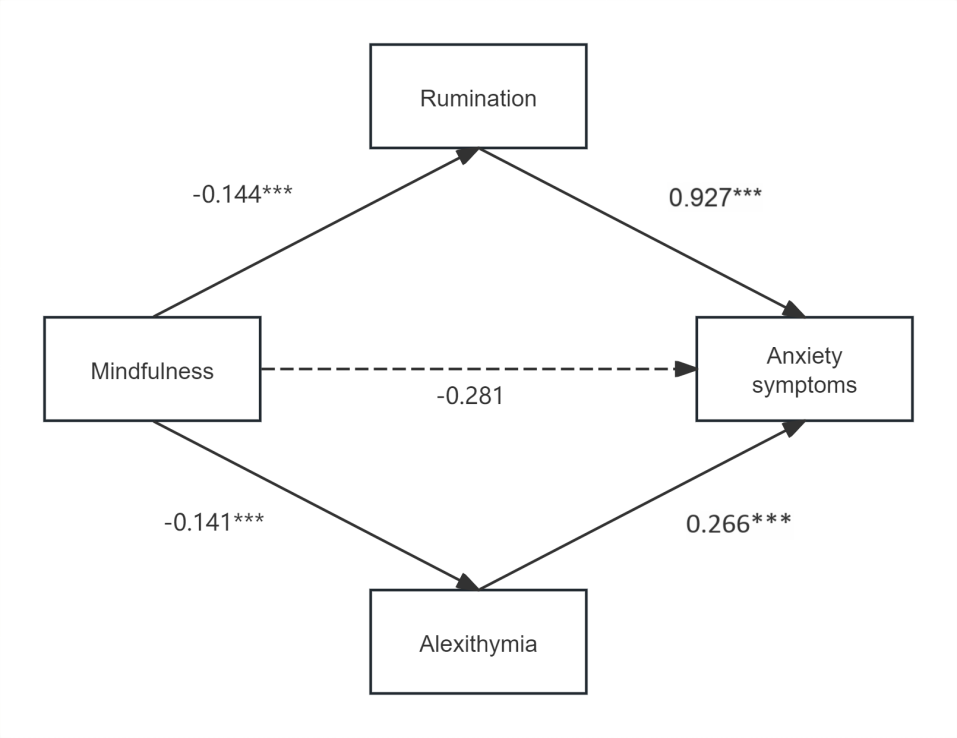


**Supplementary Fig. S2.** Parallel Mediation Pathways

Note. N = 860. Standardized regression coefficients derived from PROCESS Model 4. Solid paths indicate significant mediation effects (***p < .001). The model demonstrates parallel mediation through rumination and alexithymia in the mindfulness-anxiety relationship.
